# Physicochemical and Structural Characterization of Potato Starch with Different Degrees of Gelatinization

**DOI:** 10.3390/foods10051104

**Published:** 2021-05-17

**Authors:** Fen Xu, Liang Zhang, Wei Liu, Qiannan Liu, Feng Wang, Hong Zhang, Honghai Hu, Christophe Blecker

**Affiliations:** 1 Institute of Food Science and Technology, Chinese Academy of Agricultural Sciences, Key Laboratory of Agro-Products Processing, Ministry of Agriculture and Rural Affairs, Beijing 100193, China; xufen@caas.cn (F.X.); zhangliang@caas.cn (L.Z.); liuwei@caas.cn (W.L.); liuqiannan@caas.cn (Q.L.); wangfeng@caas.cn (F.W.); zhanghong03@caas.cn (H.Z.); 2 Department of Food Science and Formulation, Gembloux Agro-Bio Tech, Université de Liège, Passage des Déportés 2, B-5030 Gembloux, Belgium; christophe.blecker@uliege.be

**Keywords:** pre-gelatinization, thermal properties, rheology, structural properties, microstructure, starch gel

## Abstract

Starch gelatinization has been widely studied previously, but there is still a lack of systematical research on the relationship between the degree of starch gelatinization (DSG) and its physicochemical and structural properties. In this study, potato starch samples with DSG ranging from 39.41% to 90.56% were obtained by hydrothermal treatment. The thermal, rheological, and structural properties, as well as the water-binding capacity of samples were investigated. A starch solution with a DSG of 39.41% was partially sedimented at room temperature, while starch with a DSG of 56.11% can form a stable paste with a fine shear-thinning property, as well as samples with a DSG larger than 56.11%. The endothermic enthalpy, gelatinization range, and short-range ordered structure of starch were negatively correlated with DSG, whereas onset gelatinization temperature, apparent viscosity, and water-binding capacity were positively correlated. The viscoelasticity of starch gels was negatively correlated with the DSG after full gelatinization (DSG > 39.41%). Starch granules gradually lose their typical shape and less birefringence can be observed with increasing DSG. Hydrothermal treatment has a more significant effect on the amount of exposed hydroxyl groups than the ordered and amorphous structures of partially gelatinized starch. This study built linear correlations between starch physicochemical properties and the DSG and provided comprehensive insight into the characteristics of partially gelatinized potato starch.

## 1. Introduction

Potato starch is widely used in the food industry owing to its low cost, abundant availability, and more importantly, its unique physicochemical properties compared to other commercially available starches [1,2]. Potato starch can hydrate quickly and forms a paste with higher viscosity when heated, and a clearer gel when cooled, which differ from those of other starches such as corn, wheat, and rice [1,3,4]. The natural properties of potato starch are exploited by the food industry to provide products the required texture, appearance, density, and storage stability. However, native potato starch has some drawbacks, such as poor solubility and poor stability against heat and shear during pasting. These drawbacks have limited its application in industry. Consequently, various physical (heat-moisture treatment, annealing, pre-gelatinization, and high pressure treatment), chemical (cross-linking, substitution, acid hydrolysis, and oxidation), and genetic modification techniques have been used to modify potato starch and enhance its physicochemical properties to meet the demands of consumers [5,6,7]. Pre-gelatinization is a method widely used to modify potato starch, and pre-gelatinized starch has been extensively applied in the food industry [8,9].

Potato starch can be gelatinized in hot water by breaking the intermolecular hydrogen bond and destroying the arrangement of micelle structure in starch granules. After being fully gelatinized, the starch granules are porous with broken hydrogen bonds, birefringence disappears, and the crystalline order is lost through rapid drying at high temperature [10]. The degree of starch gelatinization (DSG) plays a very important role in imparting desirable product textures. Many starch-based food formulations do not require fully gelatinized starch for the best product characteristics. So far, most studies have been conducted using either native or fully gelatinized potato starch, and there has been a lack of attention on incomplete gelatinized potato starch.

Nevertheless, there have been studies on partial starch gelatinization induced by pre-heating for different times/temperatures or ball milling for different times. Studies reported that the pre-gelatinization treatment significantly changed the granule size distribution and rheological properties of starch dispersions [11,12,13,14]. Recent studies also showed that partially gelatinized starch with a lower DSG resulted in increased pasting viscosities and gel textural parameters, while the opposite was noted at higher DSG and native starch [15]. The partially gelatinized potato starch with different DSGs can be used as a thickener or stabilizer in many food products that receive a minimal heating process, for example, instant soups, desserts, powders in beverages, frozen noodles, and baking products [14]. The thermal, rheological, and structural characteristics of partially gelatinized starch can provide essential information for the selection of optimum processing conditions of starch and starchy foods [16]. However, systematical studies involving the physicochemical and structural properties of partially gelatinized potato starch with different DSGs and their correlations are still limited.

The objective of this study is to understand the relationship between the DSG and its physicochemical and structural properties. Thus, we prepared 14 partially gelatinized potato starch samples with relatively evenly distributed DSG data points by hydrothermal treatment. The water-binding capacity and thermal, rheological, and structural properties were analyzed, and the correlations between these physicochemical properties of starch and its DSG were established.

## 2. Materials and Methods

### 2.1. Raw Materials

Potato starch (PS) containing 89.32% starch, 0.27% protein, and 0.42% ash, based on the dry matters of samples, was purchased from Aladdin Bio-Chem Technology Co., Ltd. (Shanghai, China). Moisture content was determined by drying samples to a constant weight in a 105 °C oven (AOAC 925.09). Crude protein was determined by the Kjeldahl method, with the total nitrogen content being converted to protein content by a conversion factor of 6.25 (AOAC 979.09). Ash content was determined by dry mineralization of samples in a muffle oven at 550 °C for 8 h (AOAC 923.03), and starch content was determined by a total starch assay kit (K-TSTA; Megazyme, Ireland).

### 2.2. Sample Preparation

Deionized water was stirred in beakers at 300 rpm using a thermostatic water bath maintained at 59 ± 1 °C and 60 ± 1 °C, separately. Native starch powder was added after the deionized water reached the fixed temperature. Each batch of dispersion at a concentration of 10% (*w/w*) was thoroughly stirred for 1, 3, 6, 9, 12, 15, or 18 min, separately. Heating conditions were determined based on pre-experiments and the onset gelatinization temperature of native potato starch. The selected heating temperature provides starch samples with different DSGs in a convenient time period. Starch samples with a higher DSG are difficult to obtain when treated with a lower temperature in a relatively short time period, while starch is gelatinized too quickly when treated with a higher temperature. We therefore chose 59 °C and 60 °C as the temperatures and obtained a series of starch samples with different DSGs. After hydrothermal treatment, the beakers were quickly moved into ice water and cooled to room temperature before being put in the freezer. These partially gelatinized starch dispersions were freeze-dried for around 48 h using a vacuum freeze dryer (GenesisTM SQ, Virtis, Warminster, PA, USA) [15,17]. Starch samples were ground into powders with a mortar after drying and the resulting powder samples were sealed in plastic bags and stored in a silica-gel desiccator at 25 °C.

### 2.3. Differential Scanning Calorimetry (DSC)

The thermal properties of starch samples were investigated using a differential scanning calorimeter (DSC8000, PerkinElmer, Waltham, MA, USA). Powder samples (3 mg) were weighed into aluminum pans, and 10 μL of distilled water was added before the pans were hermetically sealed. The pans were equilibrated at room temperature for 2 h before heating from 20 °C to 100 °C at a rate of 5 °C/min [18,19]. A sealed empty aluminum pan was used as a reference. Onset temperature (T_0_), peak temperature (T_p_), conclusion temperature (T_c_), and endothermic enthalpy (ΔH) were calculated from the DSC curves using the equipment software. Gelatinization range (R) was computed as (T_c_ – T_0_), and the peak height index (PHI) was calculated by the ratio ΔH/(T_p_ – T_0_), as previously described [20]. The degree of starch gelatinization (DSG) was determined using Equation (1) given below [21,22].

DSG (%) = (1 − ΔH_sample_/ΔH_native_) × 100% (1)

where ΔH_native_ and ΔH_sample_ are the enthalpy change due to gelatinization of native and modified starches, respectively.

### 2.4. Water-Binding Capacity (WBC)

The WBC of starch samples was measured as previously described [23] with slight modifications. A suspension of 5 g of starch (dry wt. basis) in 75 mL of distilled water was agitated for 1 h at 150 rpm at 20 °C, 40 °C, 60 °C, and 80 °C, separately, and then centrifuged (3000× *g*) for 10 min. Free water was removed from the wet starch which was then continuously drained for 10 min at room temperature (20 °C). The wet starch was calculated using the difference between wet starch with tubes and empty tubes. The WBC of starch granules was calculated using the following formula:

WBC (%) = (W_2_ − W_1_)/W_1_ × 100% (2)

where W_1_ and W_2_ are the dry weight of original starch and wet weight of starch, respectively.

### 2.5. Fourier Transform Infrared (FTIR) Spectroscopy

The short-range molecular order of starch samples was determined using a FTIR spectrometer (Tensor 27, Bruker Opticals Company, Rheinstetten, Germany). FTIR spectra were recorded from 4000 to 600 cm^−1^ at a resolution of 4 cm^−1^ with an accumulation of 64 scans using the attenuated total reflectance (ATR) accessory. All spectra were baseline corrected automatically by OMNIC 8.2 and deconvoluted from 1200 to 800 cm^−1^ with a half-bandwidth of 19 cm^−1^ and an enhancement factor of 1.9. Intensity measurements were performed on the deconvoluted spectra by recording the peak height of absorbance bands from the baseline. The ratio of absorbance at 1047/1022 cm^−1^ and 1022/995 cm^−1^ was used to estimate the short-range ordered structure of starch [6,24].

### 2.6. Scanning Electron Microscopy (SEM)

The microstructures of the starch samples were observed using field-emission environmental scanning electron microscopy (SU8010, Hitachi, Tokyo, Japan) with a 10-KV acceleration voltage. Powder samples were added to double-sided adhesive tape mounted on an aluminum stub and sprayed with gold. Samples were photographed at 500× and 1000× magnification.

### 2.7. Polarized Light Microscopy (PLM)

The birefringence of starch samples was observed under polarized light with a binocular microscope (BA310 Pol, Motic, Xiamen, China) at a magnification of 100×. The starch samples were transferred onto a slide glass and a drop of water was then added to each sample before observation.

### 2.8. Rheological Measurement

The rheological properties of the starch samples were determined using a rheometer (Physica MCR301; Anton Paar, Graz, Austria). Starch suspensions at 10% (*w/w*) concentration after hydrothermal treatment (as described in the Section 2.2) were loaded onto the measuring apparatus after rapid cooling to room temperature. The 5 cm in diameter parallel-plate geometry with a 1 mm gap was used for all the measurements. The outer edge of the sample was coated with silicone oil to minimize water loss during measurements [23]. Temperature was controlled using a water bath system connected to a Peltier system in the bottom plate to accurately control temperature during rapid heating and cooling.

#### 2.8.1. Steady Shear Viscosity Measurement

Steady shear tests were programmed to increase the shear rate from 0.01 s^−1^ to 100 s^−1^ with six points per decade. Apparent viscosity values were obtained as a function of shear rate. The flow behaviors of mixed starch samples were analyzed using a power law equation [25,26].

(3) $$\sigma =K\cdot \gamma^{n}$$

where σ (Pa) is the shear stress, K (Pa s^n^) is the consistency index, γ (s^−1^) is the shear rate, and n is the flow index [26].

#### 2.8.2. Temperature Sweep

In the temperature sweep test, the strain and frequency were set at 0.5% and 1 Hz, respectively (within the linear viscoelastic region). The temperature was set from 25 °C to 75 °C at a heating rate of 2 °C/min and cooled from 75 °C to 25 °C at a rate of 5 °C/min. The storage modulus (G′), loss modulus (G″), and loss tangent (tanδ = G″/G′) were obtained as a function of temperature [27].

#### 2.8.3. Frequency Sweep

Frequency sweep tests were conducted in situ after the temperature sweep. Mechanical spectra of starch gel samples were recorded in the range of 0.1–100 rad/s with 10 points per decade, and the controlled variable was set at 5% strain (within the linear viscoelastic region) [26,28]. All frequency sweep tests were performed at 25 °C. G′, G″, and tanδ were obtained by changing the frequency. The power law models represented in Equations (4) and (5) were applied to describe the frequency dependence of G′ and G″, respectively.

G′ = K′∙ω^n′^ (4)

G″ = K″∙ω^n″^ (5)

In Equations (4) and (5), K′, K″, n′, and n″ are the corresponding fitting parameters, respectively, and ω is the angular frequency (Hz). K′ and K″ reflect the elastic and viscous properties, respectively. n′ and n″ are referred to as the frequency exponents and can provide useful information regarding the viscoelastic nature of materials [25].

### 2.9. Correlation Analysis

The physicochemical properties of potato starch that depend on its DSG were analyzed. Scatter diagrams were plotted, and the linear variation regression equation and correlation coefficient were calculated using Excel. SPSS was used to conduct the bivariate correlation test of the two datasets; the P value was obtained to determine the significance level of linear correlation between the starch properties and the DSG.

### 2.10. Statistical Analysis

Data are reported as averages of triplicate observations and expressed as the mean ± standard deviation. ANOVA followed by Tukey’s test at the 0.05 significance level was used to evaluate the differences among sample means. ANOVA was conducted using SPSS 18.0 (SPSS Inc., Chicago, IL, USA). All graphs were generated using Excel and Origin 9.

## 3. Results and Discussion

### 3.1. Thermal Properties

**Figure 1:**
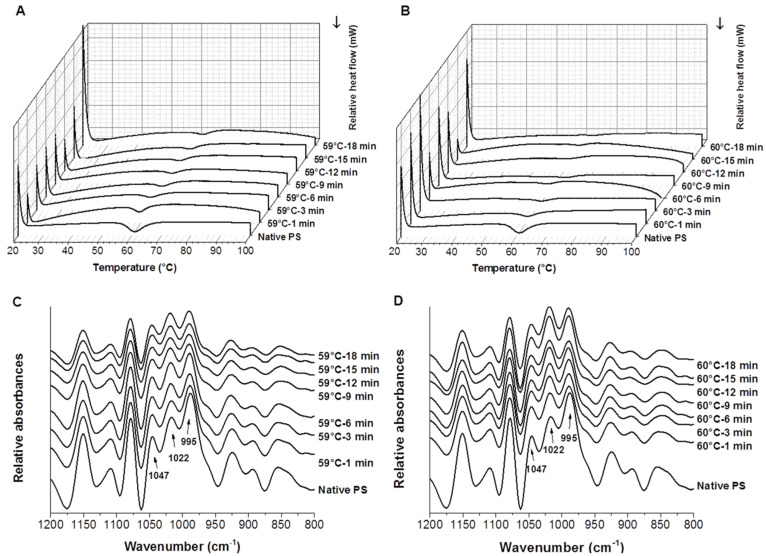
DSC thermograms (**A**,**B**) and FTIR spectra (**C**,**D**) of native and partially gelatinized potato starch samples (**A**,**C**: pre-heated at 59 °C; **B**,**D**: pre-heated at 60 °C).

The DSC thermograms in Figure 1A,B clearly show the phase transition peaks of starch samples, and as heating time increased, there was an obvious decrease in the areas under the thermal transition peak above the extrapolation, and the curves tend to be flat. The DSC parameters including T_0_, ΔH, R, and PHI are shown in Table 1. The T_P_ and T_c_ of the starch samples showed no significant differences (data not shown). The T_0_ and ΔH of the native potato starch samples were 56.94 °C and 12.73 J/g, respectively, which were values close to previous reports [29,30]. The T_0_ of the partially gelatinized starch samples was higher than that of native potato starch, and it increased as the heating time and temperature increased. The gelatinization temperature of the partially gelatinized starch modified by spray drying or high hydrostatic pressure was also reported to be higher than that of the native starch [13,31]. The increasing DSC thermal transition temperature of the heat-gelatinized starch could be due to the colloidal molecular structure of the starch granules, the amylopectin chain length, and the reordering of the crystalline structure after hydrolysis [31]. Specifically, a previous study reported that the increasing gelatinization temperature of pre-heating starch was interpreted as being due to the disruption of less stable crystallites in the first instance, followed by the melting of the remaining and more stable crystallites at a higher temperature [15,32]. However, partially gelatinized starch modified by ball milling showed the opposite variation tendency [12]. The difference might be due to the different residual crystalline structure of partially gelatinized starch after various modifications. On the contrary, ΔH, R, and PHI decreased as the heating time and temperature increased. Table 1 also shows that the calculated DSG of partially gelatinized potato starch varied from 39.41% to 90.56%. As the heating time and temperature increased, the DSG of the starch samples increased. Although the DSG of the starch sample heated at 59 °C for 9 min was slightly higher than the sample heated for 12 min, no significant difference was observed between them. The thermal parameters are known to depend on the stability of the amorphous and crystalline regions of starch [33]. Specifically, thermal parameters depend on the thickness of crystals, their polymorphic structure, and the free energy of the surface of the face side [34]. Samples with higher heating temperatures and longer heating times decreased the crystalline regions and the crystal strength within a starch granule, thus requiring less energy for full gelatinization. This may explain the decrease of R, PHI, ΔH, and the increase of DSG of potato starch as the heating time and temperature increased.

### 3.2. Water-Binding Capacity (WBC)

Table 2 shows the WBC of starch samples under 20 °C, 40 °C, 60 °C, and 80 °C. Results indicated that the WBC of all partially gelatinized starch samples was higher than that of the native potato starch. With increased pre-heating time, the WBC of starch samples significantly increased. Moreover, a significant increase in the WBC value can be observed when the reheating temperature increased from 20 °C to 60 °C. However, the WBC did not significantly increase when the reheating temperature further increased from 60 °C to 80 °C. Similarly, a previous study on pre-gelatinized rice starch showed that the water absorption index of modified samples was significantly higher than that of native starch due to the disruption of crystalline structure and the gelatinization of starch [17]. The differences in the WBC of potato starch samples may be attributed to variation in their granular structures [27]. The loose association of amylose and amylopectin molecules in starch granules is responsible for high WBC [35]. The formation of hydrogen and covalent bonds between starch chains engaged by hydroxyl groups could reduce the WBC, and differences in the availability of water-binding sites may also lead to changes in the WBC [36]. In this case, the crystalline structure of samples may be (partially) disrupted due to the breakage of intra- and intermolecular hydrogen bonds when the starch is pre-heated in excess water. The hydroxyl groups of amylose and amylopectin were exposed to a certain extent, potentially forming hydrogen bonds with water molecules and, thus, causing the increased WBC of starch samples with higher DSGs.

### 3.3. FTIR Spectra of Native and Partially Gelatinized Starch

The deconvoluted FTIR spectra of the native and partially gelatinized potato starch samples are shown in Figure 1C,D. FTIR spectroscopy was suggested to be sensitive to the so-called short-range order, defined as the double helical order, rather than the long-range order which is related to the packing of double helices [37]. Bands in the spectral region of 800–1200 cm^−1^ reflected changes in polymer conformation and the hydration of processed starches [38]. Bands at 1047 and 1022 cm^−1^ were associated with the ordered and amorphous structures of starch, respectively. The band at 995 cm^−1^ was mainly caused by the bending vibration of C-OH, corresponding to the hydrogen bond structure formed between the hydroxyl groups of starch macromolecules. The FTIR spectra of potato starch samples clearly show that, as pre-heating time increased, the intensities of the peak around 1022 cm^−1^ gradually increased, the peak around 1047 cm^−1^ became flatter, and the peak around 995 cm^−1^ significantly decreased. The ratio 1022/995 cm^−1^ significantly increased, while the ratio 1047/1022 cm^−1^ slightly decreased as heating temperature and time increased (Supplementary Data Figure S1). These data clearly show that higher DSGs of starch lead to the loss of the short-range ordered structure. Sevenou et al. [37] found that the ratio 1047/1022 cm^−1^ of native and fully gelatinized potato starch decreased from 1.37 cm^−1^ to 0.30 cm^−1^, and the ratio 1022/995 cm^−1^ increased from 0.40 cm^−1^ to 2.50 cm^−1^. Moreover, the variation of maximum absorbance at 995 cm^−1^ changed more significantly as a function of heating time than at 1047 cm^−1^, indicating that the hydrothermal treatment has a more significant effect on the amount of exposed hydroxyl groups than the ordered and amorphous structures of starch. The significant decrease of the peak around 995 cm^−1^ also explains the increase of the WBC of samples as the pre-heating time increased, since the band at 995 cm^−1^ mainly represents the hydrogen bond structure formed between the hydroxyl groups of starch macromolecules.

### 3.4. Morphological Properties of Native and Partially Gelatinized Starch

**Figure 2:**
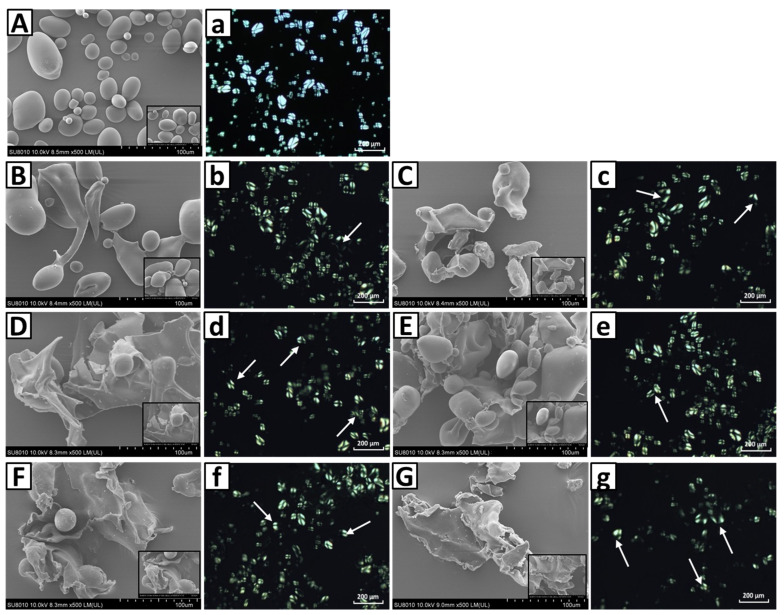
Scanning electron micrographs (upper case: **A**–**G**) and polarized light micrographs (lower case: **a**–**g**) of the native (**A**,**a**) and partially gelatinized potato starch granules (**B**–**G**,**b**–**g**) heated at 59 °C (**B**–**D**,**b**–**d**) and 60 °C (**E**–**G**,**e**–**g**) for different times (**B**,**b**,**E**,**e**: 1 min; **C**,**c**,**F**,**f**: 6 min; **D**,**d**,**G**,**g**: 18 min).

The microstructure of the native and partially gelatinized potato starch granules is presented in Figure 2. The image clearly shows that the native potato starch granules are characterized by an oval or elliptical shape with a smooth surface (Figure 2A). Starch samples heated at 59 °C and 60 °C for 1 min showed most starch granules retaining their original shape. However, with increased heating time (Figure 2B–G), the starch granules started to break and collapsed into flakes or blocks, and gradually lost their typical oval shape. The oval or elliptical shape of starch granules is largely not observed after extensive heating. The structure changes of the granules under hydrothermal treatment are mainly caused by starch gelatinization. In this process, water penetrates the starch granules and initiates irreversible swelling and microcrystal melt [9]. The polarized light micrographs showed that native potato starch granules have strong birefringence patterns (Figure 2a), reflecting a high degree of ordered molecular orientation and average radial orientation of helical structures in native potato starch [39]. The partial gelatinization treatment resulted in the disappearance of birefringence in potato starch granules to varying degrees. Compared with native potato starch sample, the partially gelatinized starch samples showed larger voids at the granule center. The loss of radial orientation in partially gelatinized potato starch granules is mainly caused by increased mobility of starch chains at the granule center following hydrothermal treatment. More thermal energy was imparted to starch chains during heating and resulted in the destruction of more molecules double helix structure; thus, less birefringence was observed.

### 3.5. Rheological Properties of Native and Partially Gelatinized Starch

#### 3.5.1. Apparent Viscosity

**Figure 3:**
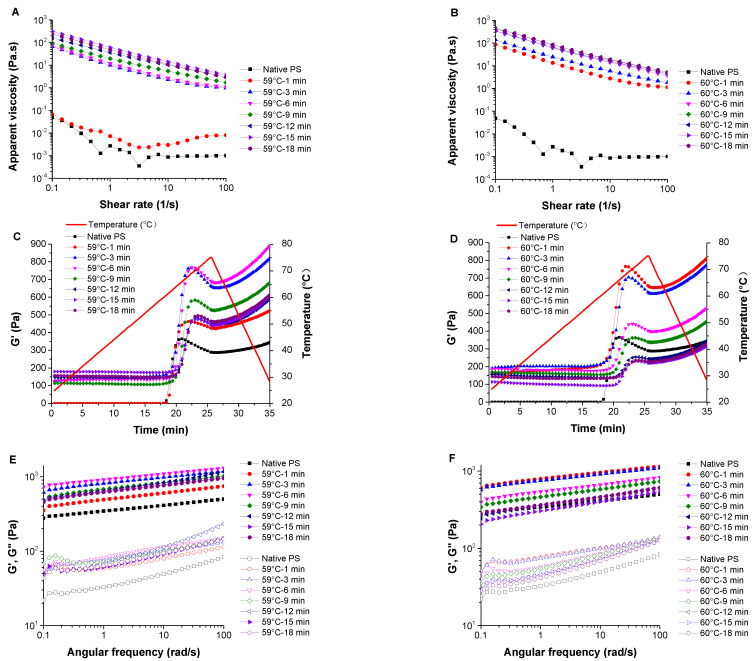
The apparent viscosity flow (**A**,**B**), temperature dependence of G′ (**C**,**D**), and frequency dependence of G′ (solid symbols) and G″ (open symbols) (**E**,**F**) of native and partially gelatinized potato starch samples (**A**,**C**,**E**: pre-heated at 59 °C; **B**,**D**,**F**: pre-heated at 60 °C).

The flow curves of starch solutions/pastes are presented in Figure 3A,B. The apparent viscosity curves decreased as the shear rate increased, and the steady shear curves were almost parallel to one another, except for the native starch solution and starch sample pre-heated at 59 °C for 1 min. This result indicated that these samples exhibited shear-thinning behavior, albeit at different levels. The shear-thinning behavior of starch samples is attributed to the destroying of the molecular network in starch pastes due to applied shear strain [9]. Similarly, shear-thinning behavior was also observed in pre-heated Peruvian carrot starch and cross-linked waxy maize starch dispersions [25,40]. Table 3 shows the power-law fitting parameters of the steady flows. Data for the native starch solution and starch sample pre-heated at 59 °C for 1 min (DSG = 39.41%) are not shown because these two samples failed to form uniform pastes, were unstable, and sedimentation occurred during the rheology measurements; thus, they do not fit the power law model. Partially gelatinized potato starch with a DSG of 56.11% can form a stable paste with a fine shear-thinning property, as well as starch samples with a DSG larger than 56.11%. Equation (3) can nicely fit the rest of the potato starch flow curves with R^2^ between 0.997 and 0.999. The consistency index (K) of the starch pastes increased significantly with increased heating time and temperature. This indicated that starch pastes with higher DSGs showed higher apparent viscosity. All flow behavior indexes (n) were less than 1.0, further indicating the pseudoplastic and shear-thinning behavior of the samples [41]. The n value increased with the heating time and temperature, indicating that starch samples with higher DSGs showed reduced pseudoplasticity. Pseudoplasticity can be attributed to the progressive orientation and alignment of the starch molecules, and the breaking of hydrogen bonds formed among amylose molecules under the influence of the shear field [42]. A high degree of macromolecular disorganization enhanced the solubility of gelatinized potato starches, leading to the formation of viscous pseudoplastics [43].

#### 3.5.2. Temperature Sweep

Figure 3C,D shows the temperature dependence of G′ of starch samples. Apart from the native starch sample and partially gelatinized starch samples pre-heated for 1 min at 59 °C, the peak G′ of other starch samples decreased as the heating time increased. This indicated that the elasticity modulus of partially gelatinized starch decreased at the re-gelatinization process with its DSG. The hydrogen bonds between starch molecules of the partially gelatinized starch samples were broken to some extent in the pre-gelatinization process, thus decreasing its modulus in the reheating process. For the native potato starch samples, starch granules first gradually swelled and had a relatively high interaction between starch molecules, requiring more energy to break the bond. This may primarily explain why the peak G′ decreased with the increased DSG. Tanδ against temperature is used to identify the gelatinization onset in oscillatory tests by the temperature at the maximum point of Tanδ [44]. As the heating time increased, the gelatinization onset temperature of the samples pre-heated at 59 °C increased from 58.4 °C to 65.9 °C, and that of the samples pre-heated at 60 °C increased from 60.13 °C to 67.90 °C (Supplementary Data Figure S2 and Table S1). This phenomenon indicated that higher degrees of starch gelatinization delayed the re-gelatinization process. This might be due to the disruption of less stable crystallites in the first instance, followed by the melting of the remaining more stable crystallites at the higher temperature [15,32]. The retrogradation may also influence the gelatinization temperature of partially gelatinized starch by forming different crystalline structures. The results are consistent with the onset temperature (T_0_) obtained from the DSC measurements.

#### 3.5.3. Frequency Sweep

Frequency sweep tests were performed on the starch gels formed in situ after the temperature sweep. The G′ and G″ of the samples were dependent on the oscillation frequency, indicating a typical viscoelastic nature of the starch gels [42] (Figure 3E,F). G′ and G″ increased as the frequency increased; therefore, the overall chain mobility within the network was relatively high. G′ was higher than G″ at the same angular frequency throughout the entire tested angular frequency range (0.1 to 100 rad/s), indicating that these starch gels exhibited dominant elastic behavior compared to viscous behavior [45]. The power-law fitting parameters affected by the frequency are shown in Table 3. The power law model represents the experimental data of G′ and G″ since the determination coefficients (R′^2^ and R″^2^) of G′ and G″ were all above 0.86. The K′ of all starch gel samples was higher than K″ at the same angular frequency, consistent with the changes of G′ and G″. K′ and K″ decreased with the increase of pre-heating time, indicating that the viscoelastic moduli of starch gels decreased with increasing DSG after full gelatinization. The tanδ of starch gel samples ranged from 0.06 to 0.24 (Supplementary Figure S2). Similarly, a previous study reported starch films exhibiting a tanδ from 0.05 to 0.25 [45]. Moreover, as a dimensionless parameter, tanδ increased with increased pre-heating time, indicating the viscous nature was dominant over the elastic nature. The pre-gelatinization treatment under certain conditions (forming uniform starch pastes with a low DSG) can provide the starch gels relatively high viscoelasticity after full gelatinization. The n′ and n″ values of all starch gels were very similar, indicating that these samples have similar frequency sensitivity.

### 3.6. Correlation between the DSG and Its Physicochemical Properties

**Figure 4:**
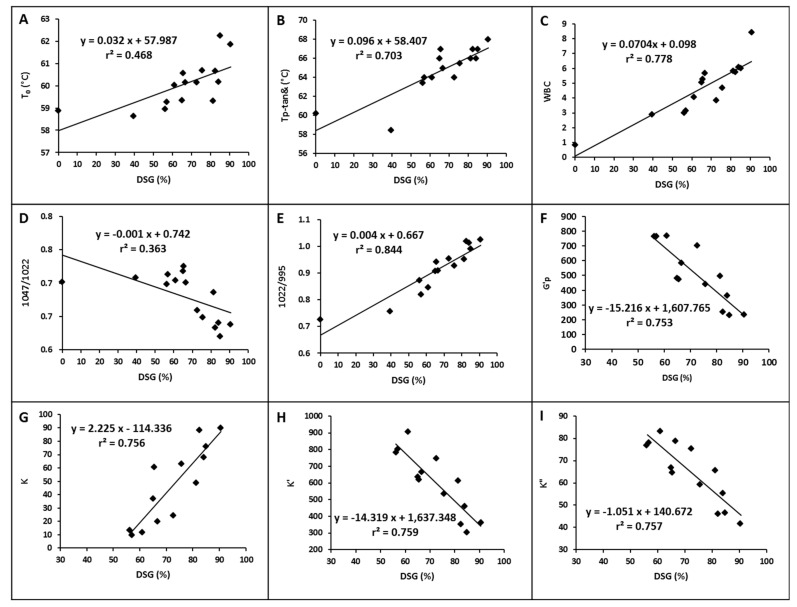
Relationship between the physicochemical properties of potato starch and its degree of gelatinization (**A**: T_0_ from DSC; **B**: temperature at the peak tanδ from temperature sweep; **C**: WBC at 20 °C; **D**: ratios of absorbance 1047/1022 cm^−1^ from FTIR; **E**: ratios of absorbance 1022/995 cm^−1^ from FTIR; **F**: peak G′ from temperature sweep; **G**: K value from steady shear test; **H**: K′ value from frequency sweep; **I**: K″ value from frequency sweep).

The physicochemical properties of potato starch as a function of the DSG are shown in Figure 4. Results showed a linear dependent relation between the starch properties and DSG, with their corresponding coefficients of determination (r^2^) differing. The prominence detection of the two datasets analyzed was less than 0.01, indicating that these starch properties were significantly correlated with the DSG when the significance level was below 0.01. Both the T_0_ from the DSC test and the temperature at peak tanδ from the temperature sweep of the rheometer were positively correlated with the DSG (Figure 4A,B), indicating that the gelatinization temperature of partially gelatinized potato starch increased in the reheating process as the DSG increased. The WBC and K values were also positively correlated with the DSG (Figure 4C,G). The ratios of absorbance 1047/1022 cm^−1^ were negatively correlated with the DSG, while the ratios 1022/995cm^−1^ were positively correlated with the DSG (Figure 4D,E), confirming the loss of the short-range ordered structure of the starch samples with a higher DSG. Moreover, the r^2^ of 1022/995cm^−1^ with DSG was 0.844, which is much higher than that of the 1047/1022cm^−1^ with DSG (0.363). Thus, the value of 1022/995 cm^−1^ from the FTIR better represents the DSG of partially gelatinized potato starch after hydrothermal treatment. The maximum point of G′ in the reheating process, K′, and K″ of potato starch samples with DSGs higher than 39.41% were also negatively correlated with their DSG, indicating that the viscoelasticity of starch gels decreased with increasing DSG (Figure 4F,H,I).

## 4. Conclusions

Native and partially gelatinized potato starch (DSG = 39.41%) cannot form uniform pastes in cold water (10%, *w*/*w*). However, partially gelatinized potato starch with a DSG of 56.11% can form stable paste with a fine shear-thinning property, as well as starch samples with a DSG larger than 56.11%. The apparent viscosity, WBC, and gelatinization onset of partially gelatinized potato starch increased as the DSG increased. Higher starch DSG led to the loss of the short-range ordered structure, and we also found that hydrothermal treatment has a more significant effect on the amount of exposed hydroxyl groups than the ordered and amorphous structures of starch. Partially gelatinized potato starch prepared under certain conditions (form uniform starch pastes in cold water with a low DSG) could form starch gels with relatively high viscoelasticity after reheating. The loss of oval or elliptical structures and the disappearance of birefringence of the starch granules can be clearly observed with increasing DSG. A correlation analysis between the starch physicochemical properties and the DSG further confirmed their linear relationships. The findings are expected help researchers better design the processing conditions for partially gelatinized potato starch and to provide information to better formulate food systems with added potato starch. However, since a heating process is normally required in food processing, further study is still needed to investigate the gelatinization and retrogradation of partially gelatinized potato starch with different DSGs during the reheating process. The molecular mechanism leading to the corresponding gelling behaviors still needs to be revealed.

## Figures and Tables

**Table 1:** DSC parameters of native and partially gelatinized starch samples.

Starch	T_0_ (°C)	ΔH (J/g)	R	PHI	DSG (%)
Native PS	56.94 ± 0.06 ^BCde^	12.73 ± 0.09 ^Aa^	8.46 ± 0.06 ^Dbc^	3.2 ± 0.01 ^Aa^	0
59 °C—1 min	56.31 ± 0.06 ^C^	6.56 ± 0.43 ^B^	9.49 ± 0.42 ^C^	2.32 ± 0.11 ^B^	39.41 ± 3.98 ^C^
59 °C—3 min	56.86 ± 0.45 ^BC^	4.66 ± 0.81 ^C^	11.02 ± 0.33 ^AB^	1.39 ± 0.42 ^C^	56.93 ± 7.50 ^B^
59 °C—6 min	57.40 ± 0.37 ^B^	4.97 ± 0.33 ^C^	11.41 ± 0.04 ^A^	1.54 ± 0.12 ^C^	60.99 ± 2.59 ^B^
59 °C—9 min	57.61 ± 0.58 ^B^	4.26 ± 0.02 ^C^	10.88 ± 0.58 ^AB^	1.31 ± 0.23 ^C^	66.56 ± 0.12 ^B^
59 °C—12 min	56.99 ± 0.41 ^BC^	4.46 ± 0.37 ^C^	10.31 ± 0.54 ^BC^	1.24 ± 0.12 ^CD^	64.98 ± 2.88 ^B^
59 °C—15 min	57.42 ± 0.40 ^B^	4.40 ± 0.43 ^C^	11.08 ± 0.30 ^AB^	1.37 ± 0.14 ^C^	65.43 ± 3.39 ^B^
59 °C—18 min	59.05 ± 0.25 ^A^	2.38 ± 0.84 ^D^	9.60 ± 0.34 ^C^	0.79 ± 0.19 ^D^	81.28 ± 6.60 ^A^
60 °C—1 min	56.50 ± 0.18 ^e^	5.59 ± 0.19 ^b^	12.46 ± 0.03 ^a^	1.38 ± 0.09 ^b^	56.11 ± 1.52 ^d^
60 °C—3 min	58.40 ± 0.25 ^c^	3.50 ± 0.16 ^c^	10.82 ± 1.77 ^ab^	0.96 ± 0.10 ^c^	72.55 ± 1.24 ^c^
60 °C—6 min	57.38 ± 0.23 ^d^	3.10 ± 0.13 ^cd^	11.45 ± 0.08 ^a^	0.86 ± 0.02 ^cd^	75.63 ± 1.01 ^bc^
60 °C—9 min	60.26 ± 0.15 ^a^	2.03 ± 0.02 ^de^	11.24 ± 0.98 ^a^	0.55 ± 0.03 ^ef^	84.06 ± 0.15 ^ab^
60 °C—12 min	57.36 ± 0.19 ^d^	2.25 ± 1.32 ^de^	10.59 ± 0.98 ^ab^	0.65 ± 0.28 ^de^	82.37 ± 10.38 ^abc^
60 °C—15 min	59.15 ± 0.01 ^b^	1.92 ± 0.39 ^de^	6.84 ± 1.15 ^c^	0.65 ± 0.12 ^de^	84.94 ± 3.04 ^ab^
60 °C—18 min	60.54 ± 0.58 ^a^	1.20 ± 0.03 ^e^	9.83 ± 1.71 ^ab^	0.33 ± 0.03 ^f^	90.56 ± 0.20 ^a^

Data are means ± SD. ^A, B, C, D.^ represent the significant difference of starch samples in column by heating at 59 °C (*p* < 0.05); ^a, b, c, d, e, f^ represent the significant difference of starch samples in column by heating at 60 °C (*p* < 0.05).

**Table 2:** The WBC of native and partially gelatinized starch samples (10^−2^%).

Starch	20 °C	40 °C	60 °C	80 °C
Native PS	0.85 ± 0.02 ^Efb′^	1.34 ± 0.17 ^Adb′^	7.74 ± 0.60 ^Dca′^	7.11 ± 0.21 ^Dca′^
59 °C—1 min	2.88 ± 0.03 ^Dd′^	4.14 ± 0.34 ^Bc′^	10.45 ± 0.02 ^Ca′^	9.43 ± 0.61 ^Cb′^
59 °C—3 min	3.15 ± 0.02 ^Dc′^	4.74 ± 0.05 ^Bb′^	13.70 ± 0.27 ^Aba′^	13.52 ± 0.04 ^Ba′^
59 °C—6 min	4.05 ± 0.13 ^Cc′^	6.14 ± 0.32 ^Ab′^	13.76 ± 0.63 ^Aba′^	14.77 ± 0.36 ^Aa′^
59 °C—9 min	5.67 ± 0.28 ^Ac′^	6.57 ± 1.24 ^Ab′^	14.42 ± 0.47 ^Aa′^	15.02 ± 0.14 ^Aa′^
59 °C—12 min	5.05 ± 0.15 ^Bb′^	6.25 ± 0.01 ^Ab′^	13.19 ± 0.63 ^Ba′^	14.30 ± 0.58 ^Aba′^
59 °C—15 min	5.29 ± 0.45 ^ABc′^	6.52 ± 0.59 ^Ab′^	14.80 ± 0.07 ^Aa′^	14.93 ± 0.08 ^Aa′^
59 °C—18 min	5.81 ± 0.40 ^Ab′^	6.58 ± 0.14 ^Ab′^	14.14 ± 0.60 ^Aba′^	14.76 ± 0.41 ^Aa′^
60 °C—1 min	3.01 ± 0.05 ^ed′^	4.33 ± 0.10 ^cc′^	13.63 ± 0.06 ^ba′^	12.83 ± 0.42 ^bb′^
60 °C—3 min	3.85 ± 0.20 ^dd′^	5.23 ± 0.02 ^bcc′^	13.66 ± 0.78 ^bb′^	15.25 ± 0.19 ^aa′^
60 °C—6 min	4.67 ± 0.12 ^cc′^	6.26 ± 0.24 ^bb′^	14.24 ± 0.34 ^aba′^	14.87 ± 0.19 ^aa′^
60 °C—9 min	6.08 ± 0.18 ^bc′^	8.13 ± 0.33 ^ab′^	14.78 ± 0.01 ^aa′^	15.09 ± 0.24 ^aa′^
60 °C—12 min	5.76 ± 0.89 ^bc′^	7.88 ± 0.50 ^ab′^	14.73 ± 0.07 ^aa′^	14.90 ± 0.17 ^aa′^
60 °C—15 min	6.02 ± 0.30 ^ab′^	8.84 ± 1.16 ^ab′^	14.90 ± 0.06 ^aa′^	15.02 ± 0.23 ^aa′^
60 °C—18 min	8.42 ± 0.09 ^bc′^	8.91 ± 0.13 ^ab′^	14.92 ± 0.07 ^aa′^	15.00 ± 0.01 ^aa′^

Data are means ± SD. ^A, B, C, D, E.^ represent the significant difference of starch samples in column by heating at 59 °C (*p* < 0.05); ^a, b, c, d, e, f^ represent the significant difference of starch samples in column by heating at 60 °C (*p* < 0.05); ^a′, b′, c′, d′^ represent the significant difference of starch samples heated at a different temperature (*p* < 0.05).

**Table 3:** Effect of shear rate and frequency on the consistency index (K, K′, and K″), flow behavior index (n, n′, and n″), and determination coefficient (R^2^, R′^2^, and R″^2^) of starch pastes and gels pre-heated at 59 °C and 60 °C.

Starch	K (Pa s^n^)	n	R^2^	K′ (Pa s^n^)	n′	R′^2^	K″ (Pa s^n^)	n″	R″^2^
Native PS	—	—	—	343.44 ± 38.54 ^Ec^	0.08 ± 0.01 ^Be^	0.996	33.2 ± 4.08 ^Cd^	0.19 ± 0.03 ^Abc^	0.981
59 °C—1 min	—	—	—	485.71 ± 25.19 ^DE^	0.09 ± 0.00 ^AB^	0.994	63.14 ± 7.54 ^B^	0.12 ± 0.01 ^A^	0.914
59 °C—3 min	9.74 ± 1.22 ^E^	−0.83 ± 0.06 ^C^	0.998	803.67 ± 0.92 ^AB^	0.08 ± 0.00 ^B^	0.995	78.15 ± 1.54 ^A^	0.11 ± 0.00 ^A^	0.988
59 °C—6 min	11.61 ± 0.72 ^E^	−0.81 ± 0.01 ^BC^	0.998	907.70 ± 43.09 ^A^	0.08 ± 0.01 ^B^	0.996	83.18 ± 4.51 ^A^	0.13 ± 0.01 ^A^	0.992
59 °C—9 min	19.89 ± 1.51 ^D^	−0.66 ± 0.07 ^A^	0.999	665.12 ± 11.21 ^BC^	0.09 ± 0.00 ^AB^	0.992	78.76 ± 1.74 ^A^	0.12 ± 0.00 ^A^	0.862
59 °C—12 min	37.04 ± 1.62 ^C^	−0.64 ± 0.02 ^A^	0.999	636.78 ± 160.52 ^CD^	0.12 ± 0.03 ^A^	0.990	66.98 ± 5.35 ^B^	0.23 ± 0.12 ^A^	0.980
59 °C—15 min	60.78 ± 2.83 ^A^	−0.71 ± 0.02 ^AB^	0.999	620.32 ± 63.77 ^CD^	0.10 ± 0.00 ^AB^	0.997	64.64 ± 0.88 ^B^	0.17 ± 0.01 ^A^	0.963
59 °C—18 min	48.66 ± 1.60 ^B^	−0.69 ± 0.03 ^A^	0.999	613.45 ± 24.59 ^CD^	0.10 ± 0.00 ^AB^	0.996	65.56 ± 0.67 ^B^	0.17 ± 0.01 ^A^	0.986
60 °C—1 min	13.35 ± 0.86 ^b^	−0.80 ± 0.03 ^b^	0.999	784.36 ± 48.37 ^a^	0.08 ± 0.00 ^e^	0.996	76.96 ± 5.33 ^a^	0.11 ± 0.00 ^d^	0.990
60 °C—3 min	24.45 ± 0.75 ^b^	−0.75 ± 0.01 ^ab^	0.999	747.29 ± 10.63 ^a^	0.09 ± 0.00 ^e^	0.996	75.34 ± 1.86 ^a^	0.12 ± 0.00 ^d^	0.982
60 °C—6 min	62.90 ± 1.20 ^a^	−0.72 ± 0.01 ^ab^	0.999	536.33 ± 32.29 ^b^	0.09 ± 0.00 ^de^	0.996	59.40 ± 1.97 ^b^	0.17 ± 0.01 ^c^	0.981
60 °C—9 min	67.92 ± 1.07 ^a^	−0.70 ± 0.01 ^a^	0.999	459.54 ± 29.51 ^b^	0.10 ± 0.01 ^cd^	0.998	55.44 ± 1.01 ^b^	0.19 ± 0.00 ^bc^	0.992
60 °C—12 min	88.19 ± 4.34 ^a^	−0.70 ± 0.01 ^a^	0.999	353.52 ± 62.90 ^c^	0.12 ± 0.01 ^ab^	0.997	46.16 ± 1.93 ^c^	0.21 ± 0.01 ^ab^	0.987
60 °C—15 min	76.04 ± 34.53 ^a^	−0.70 ± 0.11 ^a^	0.999	302.18 ± 25.68 ^c^	0.13 ± 0.01 ^a^	0.998	46.51 ± 0.52 ^c^	0.21 ± 0.01 ^ab^	0.993
60 °C—18 min	89.90 ± 6.80 ^a^	−0.68 ± 0.01 ^a^	0.998	361.52 ± 51.89 ^c^	0.11 ± 0.01 ^bc^	0.997	41.66 ± 2.72 ^c^	0.23 ± 0.01 ^a^	0.992

Data are means ± SD. ^A, B, C, D, E^ represent the significant difference of starch samples in column by heating at 59 °C (*p* < 0.05); ^a, b, c, d, e^ represent the significant difference of starch samples in column by heating at 60 °C (*p* < 0.05).

## Data Availability

The data presented in this study are available on request from the corresponding author.

## Supplementary Materials

The following are available online at https://www.mdpi.com/article/10.3390/foods10051104/s1, Table S1: The gelatinization onset temperatures of native and partially gelatinized potato starch samples pre-heated at 59 °C and 60 °C for different times (min) by calculating temperatures at the maximum points of Tanδ, Figure S1: Ratios of absorbance 1047/1022 cm^−1^ (open symbols) and 1022/995 cm^−1^ (solid symbols) of native and partially gelatinized potato starch samples as a function of heating time, Figure S2: Temperature (A, B) and frequency (C, D) dependence of tanδ of native and partially gelatinized potato starch samples (A, C: pre-heated at 59 °C; B, D: pre-heated at 60 °C).
