# Supplementary material for: Physicochemical and Structural Characterization of Potato Starch with Different Degrees of Gelatinization
Source: Foods. 2021 May 17;10(5):1104. doi: 10.3390/foods10051104 (PMC8156876; doi:10.3390/foods10051104)
Supplement: Supplementary file 1 [file foods-10-01104-s001.zip › foods-1173517-SI.pdf]

foods MDPI

Supplementary data

Table S1. The gelatinization onset temperatures of native and partially gelatinized potato starch samples pre-heated at 59°C and 60°C for different times (min) by calculating temperatures at the maximum points of Tanδ.

Temperature	0	1	3	6	9	12	15	18
59°C	60.13±0.58^{D}	58.40±1.21^{E}	63.90±0.00^{C}	63.90±0.00^{C}	64.90±0.00^{BC}	65.90±0.00^{AB}	66.90±0.00^{A}	65.90±0.00^{AB}
60°C	60.13±0.58^{e}	63.35±0.78^{d}	63.90±0.00^{d}	65.40±0.71^{c}	65.90±0.00^{bc}	66.90±0.00^{ab}	66.90±0.00^{ab}	67.90±0.00^{a}

Data are means ± SD. $^{A, B, C, D.}$ represent the significant difference of starch samples in row by heating at 59 °C (p<0.05); $^{a, b, c, d, e}$ represent the significant difference of starch samples in row by heating at 60 °C (p<0.05).

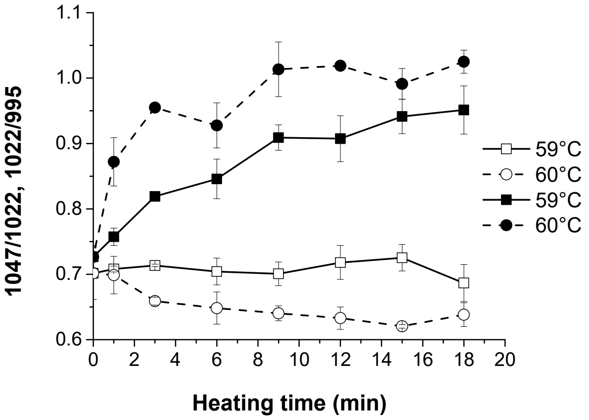

Figure S1. Ratios of absorbance 1047/1022 cm^{-1} (open symbols) and 1022/995 cm^{-1} (solid symbols) of native and partially gelatinized potato starch samples as a function of heating time.

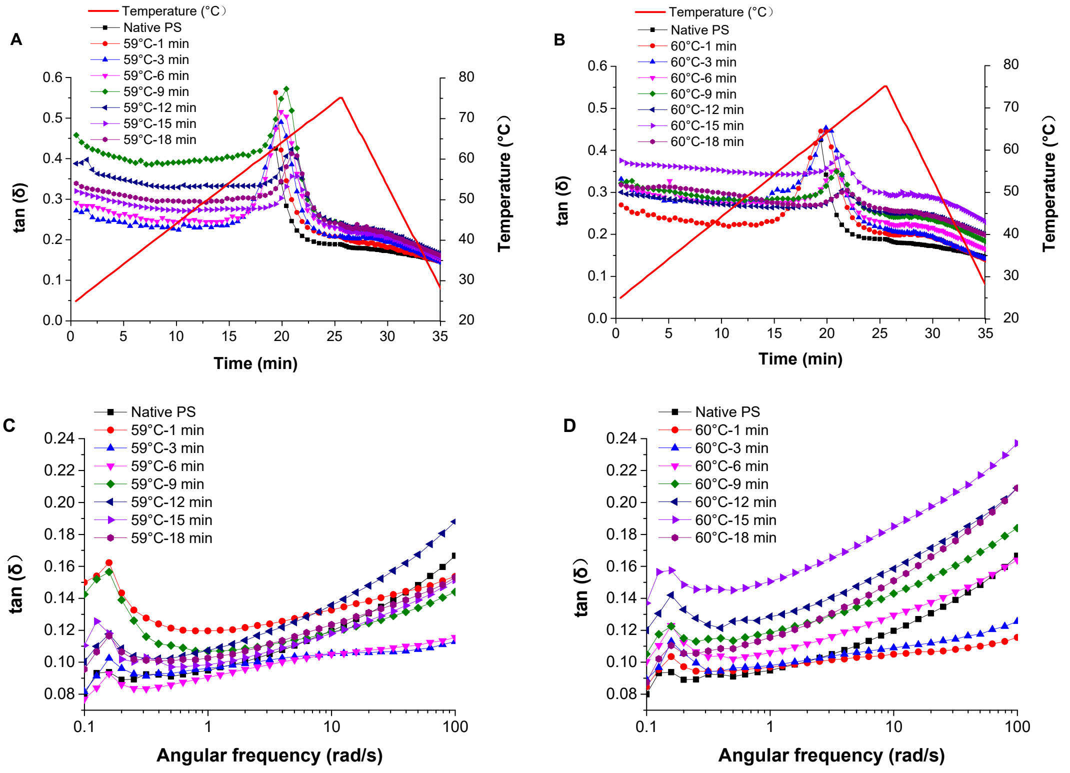

Figure S2. Temperature (A, B) and frequency (C, D) dependence of tanδ of native and partially gelatinized potato starch samples (A, C: pre-heated at 59 °C; B, D: pre-heated at 60 °C).
